# The effect of shared distinctiveness on source memory: An event-related potential study

**DOI:** 10.3758/s13415-020-00817-1

**Published:** 2020-08-24

**Authors:** Michael Weigl, Hong Hanh Pham, Axel Mecklinger, Timm Rosburg

**Affiliations:** 1 Department of Psychology, Experimental Neuropsychology Unit, Saarland University, D-66041 Saarbrücken, Germany; 2 Department of Psychology, Experimental Neuropsychology Unit, Saarland University, Campus, Building A2.4, D-66123 Saarbrücken, Germany; 3 Department of Clinical Research, Evidence-based Insurance Medicine, University of Basel, University Hospital, CH-4031 Basel, Switzerland

**Keywords:** Distinctiveness, Source memory, Illusory correlation, Subsequent memory effect, P300, Accentuation

## Abstract

An illusory correlation (IC) is the erroneous perception that two actually uncorrelated categories are correlated. The Shared Distinctiveness Approach (SDA) explains ICs with heightened accessibility of distinctive category combinations in episodic memory. However, empirical evidence for this approach is heterogeneous. In the present event-related potential (ERP) study, we exploited the fact that more distinctive items elicit larger P300 responses than less distinctive items, which potentially predict subsequent memory performance differences for such items. Distinctiveness at encoding was created by presenting words that differed from frequently presented, positive words in valence, font color, or both. We hypothesized that shared distinctiveness (deviation in both color and valence) would lead to an enhanced P300 subsequent memory effect (SME), better source memory performance, and an overestimation of the frequency of shared distinctive items. Behavioral results indicated the presence of shared distinctiveness effects on source memory and frequency estimation. Unexpectedly, memory also was enhanced for positive items in the frequent color. This pattern also was reflected in the P300 for highly positive and negative items. However, shared distinctiveness did not modulate the P300 SME, indicating that the processing of distinctive features might only indirectly contribute to better encoding. This study shows that shared distinctiveness indeed is associated with better source memory and ICs. Because effects were observed for the most frequent and the least frequent category combination, our results imply that the processing of distinctiveness might involve attention allocation to diametrical category combinations, thereby accentuating the differences between the categories.

**Electronic supplementary material:**

The online version of this article (10.3758/s13415-020-00817-1) contains supplementary material, which is available to authorized users.

## Introduction

Memory for extraordinary events often is superior to memory for ordinary events (Schmidt, 1991, 2012; von Restorff, 1933). This is not only true for dramatic events, such as 09/11, but also for more mundane ones—an unusual face, a psychologist among lawyers, or the first kiss (Hunt, 2006; Schmidt, 2012). According to Schmidt (2012), distinctiveness can arise from at least four different sources: 1) deviance from the immediate surrounding context (“primary distinctiveness”); 2) bizarreness or infrequency in lifetime experience (“secondary distinctiveness”); 3) emotionally engaging and arousing stimuli (“emotional significance”); or 4) relevant, nonarousing (“high-priority”) stimuli. All four sources have been shown to make an event more memorable (see Schmidt, 1991, for a review). Furthermore, stimuli or events become even more memorable, if they are distinctive on two or more features (Hunt & Mitchell, 1982; Kuhbandner & Pekrun, 2013; Weigl, Mecklinger, & Rosburg, 2016b). The presence of two or more distinctive features is known as shared distinctiveness or paired distinctiveness in the literature (Hamilton & Gifford, 1976; Johnson & Mullen, 1994; McArthur & Friedman, 1980).

However, distinctiveness not only affects memory, but also influences primarily memory-based frequency judgments (Tversky & Kahneman, 1973). Tversky and Kahneman (1973) proposed that availability (i.e., the ease of retrieval) of memories at the time of judgment is used to gauge the frequency of occurrence of an event in simple frequency judgments or the frequency of co-occurrence of two events in covariation judgments. Because distinctive memories are highly available, these memories affect frequency and covariation judgments (Rothbart, Fulero, Jensen, Howard, & Birrell, 1978; Tversky & Kahneman, 1973). Distinctiveness plays a particular important role in so-called illusory correlations (ICs), i.e., the subjective judgment of the covariation of two events that are actually uncorrelated (Chapman, 1967; Hamilton & Gifford, 1976).

In the first study on the IC, Chapman (1967) presented participants a series of word pairs, which contained many short words and only few long words and found that participants systematically overestimated the joint occurrence of long words. This result has been replicated in subsequent studies (Chadwick & Taylor, 2000; Tversky & Kahneman, 1973). Hamilton and Gifford (1976) extended these findings to the social domain. In their seminal study, participants read descriptions of persons who belonged either to the majority (Group A) or the minority (Group B) and showed either desirable or undesirable behavior. The majority was twice as large as the minority and desirable behavior was twice as frequent as undesirable behavior. Despite the zero correlation between group membership and desirability, the participants not only overestimated the co-occurrence of minority members and undesirable behavior, but also evaluated the minority less favorable than the majority indicating the acquisition of a group stereotype. Furthermore, the participants more accurately recalled the group membership for negative items of the minority.

Several theoretical accounts have been proposed to explain ICs, such as the memory trace model (Smith, 1991), the information loss account (Fiedler, 1991), or recurrent connectionist models (Van Rooy et al., 2003). The Shared Distinctiveness Account (SDA; Hamilton, Dugan, & Trolier, 1985; Hamilton & Gifford, 1976) is of particular importance for the present study, because we are interested in the neural underpinnings of shared distinctiveness in the IC paradigm. The Accentuation Account (McGarty et al., 1993) and the Attention Theory (AT; Sherman et al., 2009), which combines the SDA and the Accentuation Account into a unifying framework, are more relevant for the interpretation of our results than for the outline of the study or the formulation of our hypotheses. As a consequence, these accounts will be described in the discussion.

The SDA (Hamilton et al., 1985; Hamilton & Gifford, 1976) explains ICs with heightened accessibility of infrequent, distinctive category combinations in episodic memory. More precisely, the shared distinctiveness of category combinations with two infrequent attributes is supposed to lead to better encoding (relative to the more common category combinations), also increasing the availability of such distinctive category combinations at retrieval. Frequency judgments are not only determined by the actual frequency of occurrence, but also by the availability (Tversky & Kahneman, 1973). Therefore, an increased availability of rare category combinations in memory might lead to an overestimation of their frequency and, consequently, to ICs (Hamilton, 1981; Hamilton et al., 1985; Hamilton & Gifford, 1976). Indeed, studies using cued recall (see Mullen & Johnson, 1990, for a review), free recall (Hamilton et al., 1985), or one-shot ICs (Risen et al., 2007) support the heightened memory hypothesis of the SDA. However, other studies relying on signal detection theory (Fiedler et al., 1993) or multinomial processing tree models (Bulli & Primi, 2006; Klauer & Meiser, 2000) failed to accrue evidence for the SDA. To sum up, the empirical evidence for the SDA from behavioral experiments has so far been ambiguous.

In a previous behavioral study (Weigl, Mecklinger, et al., 2016b), we used group-behavior descriptions similar to Hamilton and Gifford (1976) and optimized their original design for memory tests. More precisely, the frequency of items from the majority, minority, and novel distractors in the source memory task were equated and only items from intermediate list positions were tested. This optimized paradigm not only took primacy and recency effects into account but also eliminated the confound between discrimination and response bias that usually arises from the skewed frequency distributions in the IC paradigm. The average source memory accuracy was equal for descriptions of the majority and minority. However, memory for negative behavior of the minority was elevated compared with positive behavior of the minority, even after controlling for response bias. Furthermore, source memory predicted the extent of IC.

The main goal of the present study was to reexamine the effect of shared distinctiveness on memory in the IC paradigm by using event-related potentials (ERPs). Because ERPs can be recorded online during the encoding of stimuli, ERPs can provide more direct evidence for the propositions of the SDA than behavioral measures. The P300 (also labeled P3) is an endogenous ERP component with a posterior maximum and peaks around 500 or 600 ms, when words are used as stimuli (Fabiani & Donchin, 1995; Kutas, McCarthy, & Donchin, 1977; Weigl, Ehritt, Mecklinger, & Rosburg, 2016a). The P300 is presumed to index attention allocation and has been related to the subjective probability of events (Polich, 2007).

The effect of distinctiveness on encoding processes was often investigated by the analysis of subsequent memory effects (SME; see Cohen et al., 2015; Fabiani, 2006; Friedman & Johnson, 2000, for reviews). For such an analysis, ERP data of items at encoding are retrospectively sorted depending on whether items were remembered or forgotten at testing. The analysis of SMEs can provide insights in the neural foundation of cognitive processes responsible for successful encoding (Cohen et al., 2015; Friedman & Johnson, 2000; Paller & Wagner, 2002). There is ample evidence that the P300 at encoding is related to subsequent memory performance (Fabiani & Donchin, 1995; Fabiani, Karis, & Donchin, 1990; Kamp, Bader, & Mecklinger, 2017; Karis, Fabiani, & Donchin, 1984; Neville, Kutas, Chesney, & Schmidt, 1986; Weigl, Ehritt, et al., 2016a; see Fabiani, 2006, for a review). The P300 reflects the encoding of item-specific information (Fabiani et al., 1990; Kamp et al., 2017) and the P300 SME seems to be related to subsequent recollection-based recognition, i.e., the retrieval of contextual details (Mangels et al., 2001; Weigl, Ehritt, et al., 2016a). Because distinctive events typically elicit a P300 and the P300 amplitude predicts subsequent memory, the P300 seems ideally suited for investigating shared distinctiveness in the IC paradigm.

In the present ERP study, we investigated the effects of shared distinctiveness on the P300 at encoding and on source memory by using the methodologically optimized IC paradigm, introduced in Weigl, Mecklinger, et al. (2016b). We created distinctiveness at encoding by presenting words that differed from frequently presented, positive words in valence or font color. Deviations in both—valence and font color—were considered as shared distinctiveness. As in the vast majority of IC studies (Mullen & Johnson, 1990), we chose positive items as frequent valence type. Even though studies with negative items as frequent valence type also reported ICs, the effect sizes were substantially smaller than in studies with positive items as frequent valence type (Mullen & Johnson, 1990). We hypothesized that shared distinctiveness leads to an enhanced P300 SME, better source memory, and an IC.

## Methods

### Participants

Forty healthy, right-handed students of the Saarland University (31 females; median age: 22.5 years; range: 19-30 years) participated in this study for partial course credit. The sample size for the current experiment was a-priori determined based on the effect sizes reported in the meta-analysis by Mullen and Johnson (1990) and our prior studies on the IC and ERP SMEs (Weigl et al., 2018; Weigl, Ehritt et al., 2016a; Weigl, Mecklinger, et al., 2016b). All participants were German native speakers and had normal or corrected-to-normal vision. Four additional participants had to be excluded due to lack of compliance (three participants reported napping during the experiment, and one participant took a break during an experimental task). All participants gave written, informed consent prior to participation.

### Materials

A total of 320 positive and 160 negative German nouns were selected from Lahl, Göritz, Pietrowsky, and Rosenberg (2009). Word frequency information was taken from the database dlexDB (Heister et al., 2011). Positive and negative words were matched for arousal, concreteness, word length, and word frequency. However, items could not be matched for intensity (or extremity; i.e., the valence ratings that were converted to a common scale), a factor known to affect memory for emotional words (Kamp et al., 2015). Thus, positive items were more positive than negative items were negative. The descriptive statistics for the material can be found in Table S1.

There were ten study-test cycles; 240 positive and 120 negative words were divided into ten study lists of 36 words. Each list contained 16 positive and 8 negative words presented in the majority source color and 8 positive and 4 negative words presented in the minority source color (Table 1). Purple and orange were used as font (source) colors, because prior ratings had indicated that purple and orange have the least affective or semantic connotations. For half of the participants, words in purple were the majority source and words in orange were the minority source. For the other half, the colors were reversed. The three initial words and the three words at the end of each study list (i.e., 4 positive and 2 negative words from the majority source) were items in the majority color and not used as test items in order to prevent primacy and recency effects (Weigl, Mecklinger, et al., 2016b).

**Table 1:** Distribution of positive and negative words in each study-test cycle

	Study phase		Test phase	
	Positive	Negative	Positive	Negative
Majority	16	8	8	4
Minority	8	4	8	4
New	-	-	8	4

For the test phases, 10 lists of 36 words were presented; each list contained 24 positive and 12 negative words. For the 24 positive words, 8 words each were from the majority source color, minority source color, or new. For the 12 negative words, 4 words each were from the majority source color, the minority source color, or new (Table 1).

### Procedure

**Fig. 1:**
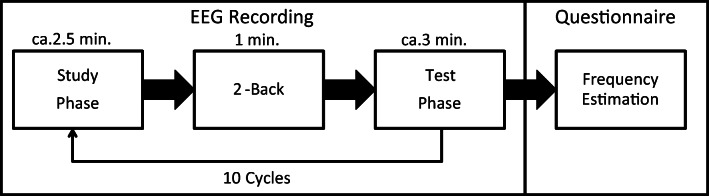
The procedure of the whole experimental session. The questionnaire with the frequency estimation task was handed to the participants only after they completed all 10 study-test cycles

The experiment consisted of ten study-test cycles (Fig. 1). Each cycle began with a study phase, followed by a distracter 2-back task, and the test phase. At the end of the experiment, participants filled out a post-experimental questionnaire.

#### Study phase

**Fig. 2:**
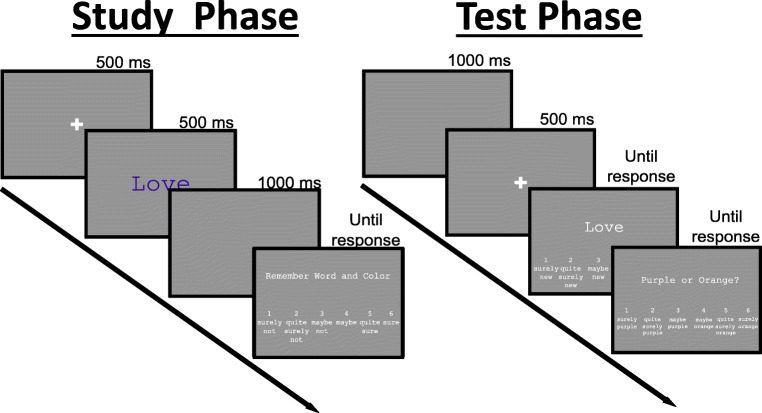
Procedure of the study phase (left) and test phase (right). Please note that the language actually used in our study was German

Each study phase trial had the following structure (Fig. 2 left): The trial began with a fixation cross presented for 500 ms. Next a word was presented either in purple (R: 128, G: 0, B: 128) or orange (R: 255, G: 165, B: 0) for 500 ms, followed by a 1,000-ms–long blank screen. Participants were instructed to remember the word and its color for a subsequent memory test. Next, participants had to make a judgment of learning (JOL; Dunlosky, Hunt, & Clark, 2000; Geraci & Manzano, 2010; Nelson & Narens, 1990), i.e., to rate how likely they would remember the word and its color on a scale from 1 (“definitely will not remember”) to 6 (“definitely will remember”). More information on the JOLs can be found in the Supplement S4. The next trial began after the response.

#### 2-back task

The 2-back task blocks had 32 trials each. Each block took roughly 1 minute to complete. Each trial had the following structure: a number between one and four was presented for 500 ms followed by a fixation cross for 1,500 ms. Participants were required to press the space bar, whenever the presented number matched the number presented two trials before. The 2-back task was introduced to prevent effects from immediate perceptual repetition and rehearsal (Grillon, Johnson, Krebs, & Huron, 2008).

#### Test phase

Each test phase trial had the following structure (Fig. 2 right): The trial began with a 1,000-ms–long blank screen, followed by a fixation cross presented for 500 ms. Next, a test cue was presented in white color, which was either a new word or a word from the study phase. The frequencies of majority, minority, and new items were equated at test, thereby reducing response bias (Weigl, Mecklinger, et al., 2016b) (Table 1). The participants had to make an old/new judgment on a six-point scale ranging from “surely new” to “surely old.” If the participants identified a word as “old,” they then had to make a judgment on the source (majority color or minority color) on a six-point scale ranging from “surely purple” to “surely orange.” There was no time limit for the old/new and source judgments. After their response, the next trial started.

#### Post-experimental questionnaire

In the post-experimental questionnaire, participants estimated the relative frequency of negative items for each source (font color) in the whole experiment in addition to several control questions about the experiment. It is common in IC research only to ask the frequency of negative items (Hamilton & Gifford, 1976; Weigl et al., 2018), because the frequency of positive items can be inferred from the participants’ responses (i.e., estimate for positive words = 1 – estimate for negative words). Frequency estimates were also separately assessed for the last block (see Supplement S5 for more information).

### EEG recording and preprocessing

An elastic cap (Easycap, Herrsching, Germany) with 58 embedded Ag/AgCl EEG electrodes was attached to the participant’s head. EEG was continuously recorded from Fp1, Fpz, Fp2, AF3, AF4, F7, F5, F3, F1, Fz, F2, F4, F6, F8, FT7, FC5, FC3, FC1, FCz, FC2, FC4, FC6, FT8, T7, C5, C3, C1, Cz, C2, C4, C6, T8, TP7, CP5, CP3, CP1, CPz, CP2, CP4, CP6, TP8, P7, P5, P3, P1, Pz, P2, P4, P6, P8, PO7, PO3, POz, PO4, PO8, O1, Oz, O2, as well as from the right mastoid (M2). We used the left mastoid (M1) as online reference and AFz as ground electrode. Electro-oculographic activity was recorded with two electrodes placed on the outer canthi and by a pair of electrodes placed above and below the right eye. Electrode impedances were kept below 5 kΩ. Data were sampled at 500 Hz and filtered online from 0.016 to 250 Hz. Only data of the study phase is reported here.

Offline, EEG data were processed with the Brain Vision Analyzer 2.0.3 (Brain Products, Gilching, Germany). Data were down-sampled to 200 Hz and a high-pass filter at 0.1 Hz was applied. Cardiovascular, muscle, and ocular artifacts were removed via independent component analysis (ICA). Next, data were re-referenced to linked mastoids and a low-pass filter at 30 Hz (48 dB/oct) was applied. Then, data were segmented into epochs of 2,200 ms (including 200 ms prestimulus baseline) and baseline correction was performed. Data were screened for remaining artifacts and all segments containing amplitudes outside the range of −100 to 100 μV or voltage steps exceeding 50 μV/ms were removed.

### Data analysis

For the analysis of source memory performance, we treated the three points “surely new,” “quite surely new,” and “maybe new” of the confidence rating as new response and the three points “surely old,” “quite surely old,” and “maybe old” as old response. A similar procedure was used for the source judgments (purple vs. orange). Thus, a correct majority source response was defined as an item that received an old response and was assigned to the majority source. Likewise, a correct minority source response was defined as an item that received an old response and was assigned to the minority source. Source memory performance was quantified by calculating the unbiased hit rates for correct source judgments (Wagner, 1993) for each source (majority, minority, new) and valence (positive, negative). Unbiased hit rates take into account both stimulus frequency and response frequency and have been used in other source memory studies (Bell et al., 2012; Suzuki & Suga, 2010; Weigl, Mecklinger, & Rosburg, 2018; see Supplement S2 for the calculation of unbiased hit rates). Unbiased hit rates are calculated by multiplying the conditional probability of correctly classifying an item as a majority, minority, or new item (i.e., p(correct response|item)) with the conditional probability of correctly applying a response category (“majority,” “minority,” or “new”) given that it is applied (i.e., p(correct response|response); see Supplement S2 for more information). The resulting values can range from 0 to 1 and thus can be interpreted like normal hit rates. The resulting values were then arcsine transformed for statistical analysis (Wagner, 1993). A Source (majority vs. minority vs. new) x Valence (positive vs. negative) repeated-measure ANOVA was calculated for the unbiased hit rates. In order to provide a more comprehensive picture of the memory performance, we also analyzed the confidence ratings in the source judgment for old items and the reaction times for correctly identified items. The confidence ratings for the source judgments were subjected to a Source (majority vs. minority) x Valence (positive vs. negative) repeated-measure ANOVA. The reaction times to the old/new judgment were subjected to a Source (majority vs. minority vs. new) x Valence (positive vs. negative) repeated-measure ANOVA.

In order to assess the extent of IC, we compared the estimated frequency of negative words for the majority and the minority source across the whole experiment using dependent t-tests. In addition, we used the frequency estimates to calculate the phi coefficient as a direct measure of the extent of IC. Fisher’s Z transformed phi coefficients were analyzed using a single-sample *t*-test.

Consistent with recommendations on best practice (Luck & Gaspelin, 2017), we restricted the ERP analysis of the P300 in the study phase to the a priori defined time window from 500 to 700 ms and the a priori selected electrode Pz. The suitability of these a priori restrictions is corroborated by the literature on the P300 SME (see Fabiani, 2006 for a review) and a prior subsequent memory study (Weigl, Ehritt, et al., 2016a). P300 SME contrasted subsequently remembered study items (i.e., items correctly judged as “old”) with subsequently forgotten study items (i.e., items falsely judged as “new”). Due to the imbalanced design resulting from the skewed frequency distribution of the IC paradigm (Hamilton & Gifford, 1976) and from the dependence of trial numbers on subsequent memory performance (i.e., high memory performance led to fewer forgotten trials and vice versa; see Table S3 for the trial numbers; Tibon & Levy, 2015), we decided to analyze the P300 on the single trial level with multilevel linear modeling (MLM; see Finch, Bolin, & Kelley, 2014, for a general introduction). MLM is an alternative to the repeated-measure ANOVA and is especially useful for unbalanced designs as in our case (Field et al., 2012). The main advantage of MLM over the repeated-measure ANOVA is that there is no need to exclude participants due to low trial numbers in specific experimental conditions and the analysis can be based on the whole sample (Tibon & Levy, 2015).

All data except the single-trial EEG analysis were analyzed using SPSS 24. Significance level was set to *p* = 0.05 for all analyses. For all repeated-measure ANOVAs, the sphericity assumption was tested with Mauchly’s test and the Greenhouse-Geisser correction was applied when necessary. For the MLM, we used R 3.4.1 and the package nlme 3.1-131 (Pinheiro et al., 2018).

## Results

### Behavioral results

#### Source memory

**Fig. 3:**
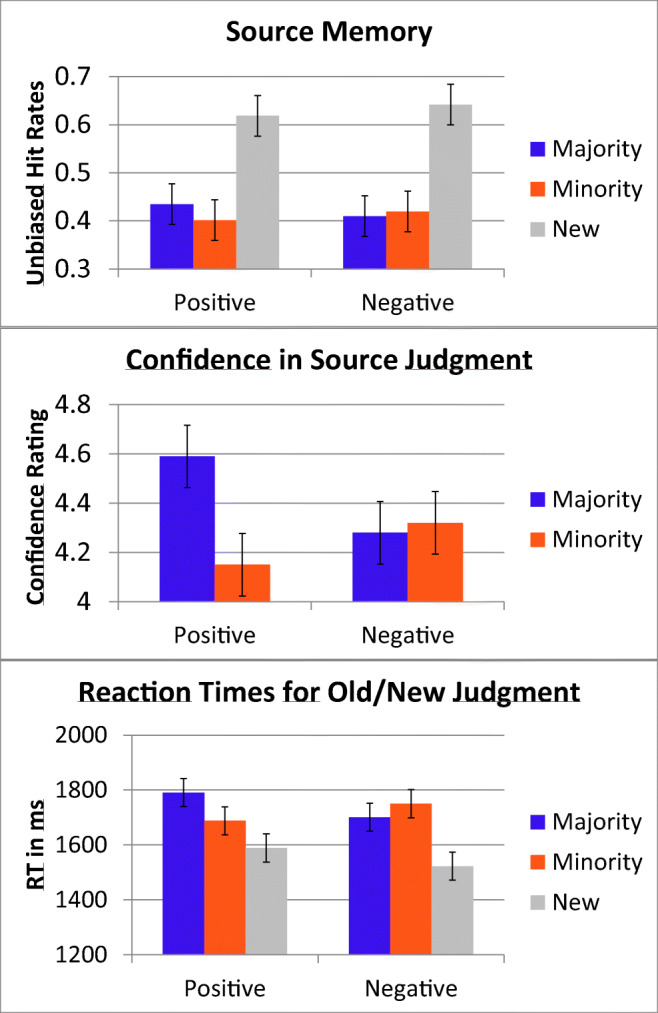
Overview over the behavioral results from the test phase. Top: Source memory performance as measured by unbiased hit rates. Middle: Source confidence ratings. Bottom: Reaction times. The error bars represent within-subject 95% confidence intervals for the source x valence interaction. Please note that the colors used for illustrating the majority/minority are exemplary: Color attribution for majority and minority words was balanced across participants

The analysis of the unbiased hit rates for correct source judgments (see Fig. 3, top) revealed a significant main effect for source (F(2, 78) = 149.67, *p* < 0.001, η_p_^2^ = 0.79), but no main effect for valence (F(1, 39) = 0.22, *p* = 0.642, η_p_^2^ = 0.01). Unbiased hit rates were higher for new items than for old items (F(1, 39) = 257.06, *p* < 0.001, η_p_^2^ = 0.87). There was no difference between the majority source and the minority source (F(1, 39) = 0.88, *p* = 0.354, η_p_^2^ = 0.02). Furthermore, there was an interaction between source and valence (F(1.69, 65.78) = 4.29, *p* = 0.023, η_p_^2^ = 0.10). The follow-up interaction contrasts for the old items was significant (F(1, 39) = 4.11, *p* = 0.050, η_p_^2^ = 0.10). The unbiased hit rates were higher in the majority than in the minority for positive items, but lower in the majority than in the minority for negative items. Follow-up *t*-tests did, however, not reveal any significant differences between positive and negative items for the majority source (t(39) = 1.55, *p* = 0.065, one-sided, Cohen’s d = 0.24) or minority source (t(39) = −1.25, *p* = 0.109, one-sided, Cohen’s d = −0.20).

The analysis of the confidence ratings for the source judgments (see Fig. 3, middle) revealed a main effect for source (F(1, 39) = 5.21, *p* = 0.028, η_p_^2^ = 0.12) and a trend for valence (F(1, 39) = 3.12, *p* = 0.085, η_p_^2^ = 0.07). There also was a significant interaction between source and valence (F(1, 39) = 10.05, *p* = 0.003, η_p_^2^ = 0.21). One-sided *t*-tests revealed that the participants were more confident in their source judgments to positive majority items than to negative majority items (t(39) = 3.70, *p* < 0.001, one-sided, Cohen’s d = 0.59), and participants were more confident in their source judgment to negative minority items than to positive minority items (t(39) = −1.99, *p* = 0.027, one-sided, Cohen’s d = −0.32).

The analysis of reaction times to items attributed to the correct source (see Fig. 3, bottom) revealed a main effect for source (F(1, 39) = 10.80, *p* < 0.001, *ε* = 0.79, η_p_^2^ = 0.22), but not for valence (F(1, 39) = 2.27, *p* = 0.140, η_p_^2^ = 0.06). Reaction times were faster for new items than for old items (F(1, 39) = 14.07, *p* = 0.001, η_p_^2^ = 0.27). No differences emerged between majority source and minority source (F(1, 39) = 0.77, *p* = 0.387, η_p_^2^ = 0.02). Critically, the interaction between source and valence was significant (F(2, 78) = 5.01, *p* = 0.009, η_p_^2^ = 0.11). Participants reacted faster to negative majority items and positive minority items than to positive majority items or negative minority items (F(1, 39) = 6.62, *p* = 0.014, η_p_^2^ = 0.15).

To sum up, a consistent pattern was observed for all three dependent variables—unbiased hit rates, source confidence ratings, and reaction times—in the source monitoring task. For the majority color, participants made more accurate and more confident, but slower memory judgments for positive items than for negative items. For the minority items, the reverse pattern was observed.

#### Frequency judgment

Across all blocks, the frequency of negative words in the minority source was overestimated relative to the frequency of negative words in the majority source (majority: M = 0.39, SD = 0.15; minority: M = 0.46, SD = 0.16; t(39) = −2.02, *p* = 0.025, one-sided, Cohen’s d = 0.32), indicating the presence of an IC. Furthermore, the participants correctly rated the majority source as highly frequent (M = 0.61, SD = 0.15; t(39) = 4.72; *p* < 0.001, one-sided, Cohen’s d = 0.75). However, their estimates were lower than the actual frequency (0.67). We also calculated a phi coefficient from the frequency ratings and found a significant IC (M = 0.07, SD = 0.21; t(39) = 2.00, *p* = 0.026, one-sided, Cohen’s d = 0.36).

Next, we ran separate multiple regressions for the unbiased hit rates and the confidence ratings to assess whether memory performance predicts the IC as proposed by the SDA. To our surprise, neither regression model was significant (unbiased hit rates: R^2^ = 0.13, F(4, 35) = 1.26, *p* = 0.303; confidence ratings: R^2^ = 0.09, F(4, 35) = 0.82, *p* = 0.521). However, the results from the regression analysis need to be treated with some caution due to violations in the assumption of homoscedasticity and of normality of the residuals.

### ERP results: P300 subsequent memory effect (500-700 ms)

#### Model selection

The P300 SME was analyzed with MLM at Pz for the time window from 500 to 700 ms. The need for a MLM was assessed with an intercept test (Field et al., 2012). For this purpose, we included participants as a random intercept (see Table 2 for information on model fit). A comparison of the random intercept model with the intercept only model revealed that the P300 amplitude at Pz varied across participants (χ^2^(1) = 541.39, *p* < 0.001; ICC = 0.07). Next, we included study block as another random intercept nested in the participants. Again, the P300 differed across study blocks (χ^2^(1) = 25.17, *p* < 0.001; ICC = 0.07 for participants, ICC = 0.02 for the blocks nested within participants). We defined this three-level model as our baseline model.^1^

**Table 2:** Information on model fit for the hierarchical linear model analyses

	AIC	BIC	Log-Likelihood
Intercept only	70159.63	70173.93	-35077.81
Random intercept for participants	69620.24	69641.70	-34807.12
Random intercept for block nested within participants (baseline model)	69597.08	69625.69	-34794.54
Model 1	69591.74	69727.65	-34776.87
Model 2	69598.17	69762.69	-34776.08

In the first analysis, we added the grand-mean centered factors Valence (Negative: −0.67, Positive: 0.33), Source (Minority: −0.50, Majority: 0.50), and Memory (Forgotten: −0.81, Remember: 0.19) and the grand-mean centered variable Intensity as well as all interactions between these variables as fixed effects to the baseline model. Intensity was included as a covariate, because the positive and negative items differed in intensity (see Section 2.2). The inclusion of these variables significantly improved the model fit (χ^2^(15) = 35.34, *p* = 0.002). Because inclusion of random slopes for Memory did not improve model fit (χ^2^(4) = 1.57, *p* = 0.813), we decided to use the random intercept model for interpretation.

#### Model interpretation

**Fig. 4:**
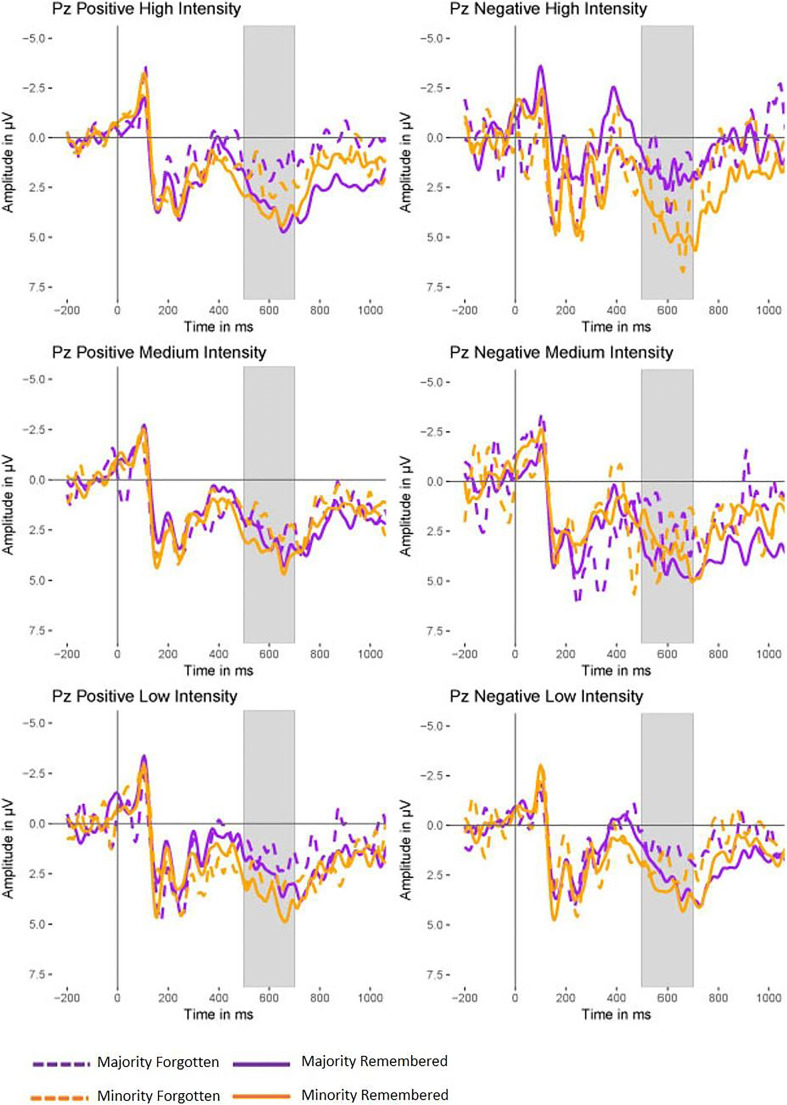
P300 at the electrode Pz in the study phase for positive and negative items of high, medium or low intensity. Dashed lines denote forgotten items and solid lines denote remembered items. The grey bar indicates the 500-700 ms time window which was used for statistical analysis. Please note that the colors used for illustrating the majority/minority are exemplary: Color attribution for majority and minority words was balanced

Information on the coefficients of the final model can be found in Table 3. The analysis revealed a significant effect for Memory indicating that the P300 was larger for subsequently remembered items compared with subsequently forgotten items (Figures S1 and S5) and a significant effect for Source indicating that the P300 was larger for the rare color than for the common color (Figures S2 and S5). There was, however, no effect for Valence (Figure S3). In contrast to our hypothesis, we failed to obtain a significant interaction between Valence and Source or between Valence, Source, and Memory. However, there were marginally significant interactions between Valence, Source, and Intensity (Figure 4) and between Intensity and Memory (see Figs. 4 and S4).

**Table 3:** Information on model fit for the hierarchical linear model analyses

	B	SE B	t(9030)
(Intercept)	3.05	0.42	7.21 (p < .001)
Valence	0.03	0.23	0.15 (p =.880)
Source	-0.76	0.21	-3.59 (p < .001)
Intensity	0.15	0.11	1.34 (p = .179)
Memory	0.96	0.28	3.41 (p < .001)
Valence x Source	0.27	0.46	0.60 (p = .547)
Valence x Intensity	-0.03	0.21	-0.13 (p = .897)
Source x Intensity	0.22	0.23	0.96 (p = .336)
Valence x Memory	-0.30	0.58	-0.51 (p = .610)
Source x Memory	-0.05	0.54	-0.09 (p = .927)
Intensity x Memory	0.58	0.29	1.96 (p = .051)
Valence x Source x Intensity	0.81	0.43	1.88 (p = .060)
Valence x Source x Memory	-0.48	1.16	-0.41 (p = .682)
Valence x Intensity x Memory	0.05	0.55	0.08 (p = .934)
Source x Intensity x Memory	0.21	0.59	0.39 (p = .700)
Valence x Source x Intensity x Memory	0.91	1.10	0.82 (p = .412)

**Fig. 5:**
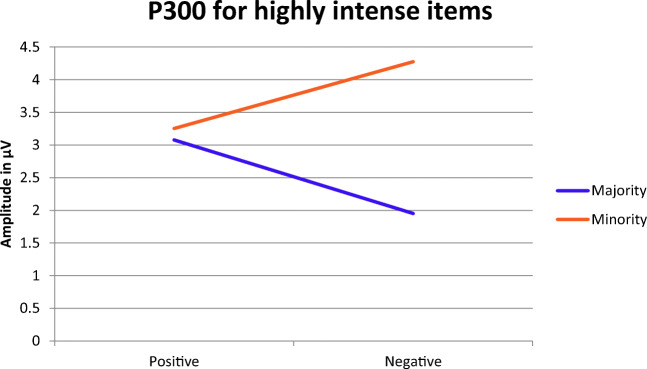
Predicted P300 amplitudes and slopes based on the follow-up analysis for highly intense items. Please note that the predicted values were calculated with the model formula (3.15 + 0.05*Valence – 0.89*Source + 2.15 * Valence * Source) and the values for Valence and Source in section 3.2.1. Please note that the colors used for illustrating the majority/minority are exemplary: Color attribution for majority and minority words was balanced

In order to follow-up these interactions, we used the terciles for Intensity to divide the data set into three subsets, a low, a medium and a high intensity subset (see Fig. 4 for the ERP waveforms). For the high intensity subset, the follow-up analyses revealed a significant interaction between Valence and Source (b = 2.15, t(2673) = 2.61, *p* = 0.009; Fig. 5). The P300 was larger for positive items than for negative items of the majority color (b = 1.15, t(1137) = 2.02, *p* = 0.044). For the minority color, a trend in the reverse direction was found, i.e., the P300 amplitudes tended to be larger for negative items than for positive items (b = −1.00, t(1138) = −1.67, *p* = 0.096). Furthermore, the P300 was significantly larger for negative items in the minority color than for negative items in the majority color (b = −2.47, t(366) = −3.26, *p* = 0.001). For positive items, however, no such difference between the majority and minority color was observed (b = −0.19, t(1990) = −0.49, *p* = 0.627). Furthermore, there also was a significant effect for Memory in the high intensity subset (b = 1.46, t(2675) = 3.19, *p* = 0.002).

The interaction between Valence and Source was not significant for the low and medium intensity subset (b = −0.54, t(2698) = −0.78, *p* = 0.434 and b = −1.06, t(2865) = −1.31, *p* = 0.189, respectively) or when collapsing across low and medium intensity items (b = −0.41, t(5966) = −0.84, *p* = 0.403). Furthermore, the effect for Memory was on a trend level for the low intensity subset (b = 0.78, t(2700) = 1.71, *p* = 0.087) and absent in the medium intensity subset (b = 0.75, t(2867) = 1.64, *p* = 0.100). However, the effect for Memory became significant when collapsing across low and medium intensity items (b = 0.76, t(5968) = 2.34, *p* = 0.019), suggesting that the SME was weaker for low and medium intensity items than for high intensity items.

To sum up, the ERP data revealed that the P300 amplitude predicted subsequent memory. The SME was most pronounced for high intensity stimuli. Furthermore, only for highly intense stimuli, shared distinctiveness affected the P300 amplitude: The P300 amplitude was larger for positive majority and negative minority stimuli than for negative majority and positive minority stimuli.

## Discussion

Most neurophysiological studies on social cognition focus on preexisting stereotypes or on person perception (Bartholow, Fabiani, Gratton, & Bettencourt, 2001) and studies on intergroup attitude formation are scant (Spiers et al., 2016). Our ERP study went a step further and distilled the core features of the IC paradigm to provide a more integrative perspective of the contribution of ICs to stereotype acquisition. To the best of our knowledge, our study is the first to investigate shared distinctiveness and ICs with ERPs.

We investigated the effect of shared distinctiveness on source memory and its neural correlates with an optimized IC paradigm introduced by Weigl, Mecklinger, et al. (2016b). Shared distinctiveness at encoding was created by presenting (frequent) positive and (infrequent) negative words, either in a frequent or infrequent color. Based on the SDA, we had hypothesized that shared distinctiveness leads to 1) better source memory for the minority than the majority and best source memory performance for negative minority items, and 2) enhanced P300 SME for the minority than the majority and the largest P300 SMEs for negative minority items, as discussed in the following.

### Effects of shared distinctiveness on memory

The prediction of the SDA for the behavioral source memory data were only partially fulfilled. Whereas source memory was indeed better for negative minority items than for positive minority items, positive majority items were better remembered than negative majority items. Moreover, overall source memory performance was actually similar for the majority and minority, contrary to the assumptions of the SDA. These behavioral findings are largely in line with some of our previous findings (Weigl, Mecklinger, et al., 2016b). The findings do not corroborate the SDA, as the shared distinctive items were not remembered best. However, in the current study, we were able to provide evidence for better memory for negative relative to positive items within the minority, when primacy and recency effects were considered and when the frequencies of majority items, minority items, and novel distractors items were equated in the source memory task. Prior IC studies, which did not consider these methodological aspects, found superior memory for the majority (Fiedler et al., 1993; Weigl et al., 2018) or for negative items (Klauer & Meiser, 2000), but not superior memory for negative items within the minority. Of note, previous research has shown that a strong memory advantage for negative minority items relative to all other conditions is more likely to be obtained, when the source memory task is replaced by free recall (Hamilton et al., 1985).

Prior studies reported reaction time facilitation for shared distinctive items relative to other category combination (Johnson & Mullen, 1994; McConnell et al., 1994). These reaction time findings provided support for the SDA, assuming that shared distinctiveness leads to high accessibility of these items in memory. Yet in our study, reaction times at test showed an opposite pattern and were faster to negative majority and positive minority items than to positive majority and negative minority items. These results seem to reflect a speed-accuracy trade-off and provide no evidence for facilitated accessibility of shared distinctive items. Thus, our reaction time findings do not support the SDA either.

Consistent with previous IC research (see Mullen & Johnson, 1990, for a review), negative minority items were estimated to be more frequent than negative majority items indicating the presence of an IC. Based on the SDA, we expected that memory performance would predict the IC. However, neither the unbiased hit rates nor the source confidence judgments predicted the extent of IC in the multiple regression analysis. This was unexpected, as prior studies investigating the relationship between memory and IC found significant correlations with memory measures (Fiedler et al. 1993; Hamilton et al., 1985; Weigl, Mecklinger et al., 2016b; Weigl et al., 2018). One potential explanation might be that the IC was smaller in the current study compared with the previous studies. However, the relationship between memory and IC was not substantiated by other studies either, such as Van Rooy et al. (2013). In this study, ICs were not contingent on the presence or absence of negative items in the least frequent group (see also Fiedler, 1991).

To sum up, our results cast doubts on the existence of a prominent direct relationship between memory for shared distinctive items and ICs, as proposed by the SDA.

### Effects of shared distinctiveness on the P300

We expected to find an enhanced P300 SME for the minority relative to the majority and the largest P300 SMEs for negative minority items. The present study revealed a pronounced generic P300 SME (Figure S1), which was, however, not modulated by shared distinctiveness. This finding does not support the notion that shared distinctiveness, as defined in our study, promotes encoding. This was surprising as prior research suggested that the P300 SME is enhanced with minority status (Weigl, Ehritt et al., 2016a) and negative valence (Kamp et al., 2015). Instead, in our study the SME increased with emotional intensity (Figure S4), suggesting that in current experimental set-up higher emotional intensity (but not source or valence) modulated encoding processing, as reflected in the P300 SME.

However, our P300 results indicate that our frequency manipulation was effective.

Consistent with the literature associating the P300 with the updating of mental schemata and subjective probability (Duncan-Johnson & Donchin, 1977; Polich, 2007), we found a larger P300 for the minority relative to the majority (Figure S2). No such effect was found for valence (Figure S3). Moreover, intensity modulated the interaction between valence and source: Only for highly intense items, P300 amplitudes were larger for negative minority items than for all other kinds of items (Fig. 5). Thus, the P300 findings suggest that minority items become distinct because of their combination of rareness, negative valence, and intensity, rather than just rareness and negative valence, as we initially presumed. This finding might be interpreted as tentative evidence for the SDA.

By contrast, the P300 was smaller for highly intense negative majority items than for highly intense positive majority items, which conflicts with the SDA (Fig. 5). The latter result cannot be attributed to misperceived frequencies for majority items, because the preponderance of the majority color and of positive items was correctly identified in the frequency estimation task. Arousal effects on the P300 can be precluded as well, because even the highly intense stimuli were still matched for arousal.

All in all, the ERP results did not provide support for the SDA either. Nevertheless, similar cross-over interaction patterns were found for memory and the P300 for highly intense stimuli. While the current results are hard to accommodate within the SDA framework, another account, namely Attention Theory, might provide a parsimonious explanation for these findings.

### Attention Theory: Shared distinctiveness, accentuation, and illusory correlations

Attention Theory (AT; Kruschke, 2003), which has been successfully applied to frequency-related phenomena like the IC (Sherman et al., 2009) offers a more integrative perspective on our results. In situations with skewed frequency distributions for categories (e.g., majority and minority) and their attributes (e.g., positive/negative valence), the speed of acquisition is higher for the frequent attribute relative to the infrequent attribute. The attribute that is learned first not only defines the category acquired first, but also what other attributes are used for differentiation and accentuation (i.e., the exaggeration of both, between-category differences and within-category similarities). In essence, AT claims that the most and the least frequent category combination in the IC paradigm receive more attention than the two remaining category combinations. This ultimately results in an IC as the majority is associated with positivity and the minority with negativity.

In our study, the positivity of the majority source should have been learned first resulting in an attention shift to negative minority items for differentiation. Consistent with this idea, we found stronger effects for positive majority and negative minority items than for the other category combinations in the behavioral measures and the P300. Thus, our results imply that attention allocated to diametrically opposed category combinations lead to the accentuation of category differences, with the P300 reflecting contrast enhancement for the most informative category combinations (i.e., majority positive and minority negative).

Although AT (Sherman et al., 2009) does not make specific predictions regarding episodic memory, it seems plausible that attention shifts contributed to better encoding of the most and the least frequent category combination. The cross-over pattern found for source memory, i.e., better source memory for positive majority and negative minority items relative to negative majority and positive minority items, are in line with this notion.

At first glance, the absence of a P300 SME modulated by valence and source and of a correlation between source memory and IC seem surprising even within the AT framework. However, neither effect is essential for AT, because attention shifts alone are enough to result in different impressions for both groups. Furthermore, AT assumes that these impressions are formed over the course of category acquisition and are not reliant on detailed episodic memory. Thus, AT can account for the main findings in our study, namely the cross-over pattern observed in memory and the P300 for highly intense items, and is not necessarily contradicted by the absence of a modulation of the P300 SME by shared distinctiveness.

To sum up, accentuation was a post-hoc explanation for the unexpected interaction pattern in the behavioral and ERP data in our study. Therefore, the accentuation interpretation has to be considered preliminary. So far, accentuation was rarely investigated in IC studies (Berndsen et al., 2001; McGarty et al., 1993) and fails to account for the presence of an IC under equated frequencies for valence (Weigl et al., 2018). Moreover, Kutzner and Fiedler (2015) fitted computational models on data from four IC experiments and showed that models without an attention shift mechanism fit the data equally well as models with such a mechanism. This indicates a clear need for more systematic investigations of accentuation and attention shifts as driving factor behind ICs. Psychophysiological methods with high temporal resolution like ERPs or eye-tracking are particularly well-suited for investigating attention shifts in the IC paradigm (Weigl, 2019).

### Caveats

Our study has several major caveats. First, there were 160 items in the most frequent category combination (i.e., positive majority items), but 200 items in the remaining three category combinations (Table 1). Thus, there were fewer items in the most frequent category combination than distinctive items. This also might have contributed to the unusual interaction pattern found in the P300. Most P300 SME studies only present a single distinctive item per list (Fabiani & Donchin, 1995; Weigl, Ehritt, et al., 2016a). A control experiment with fewer items per list in the least frequent category might provide more conclusive test of the SDA.

Second, several types of distinctiveness are inherent in the structure of the IC paradigm (Weigl et al., 2018). Primary distinctiveness was induced by the infrequency of the minority source and negative items. However, negative items are also distinctive due to secondary distinctiveness (Schmidt, 2012). Using only physical distinctiveness (i.e., size and color) might be more suitable to test the influence of shared distinctiveness on ERPs and memory than the present paradigm.

Third, given the limited range of highly positive or negative German words provided by Lahl et al. (2009), we had to include less intense words in order to achieve a high trial number and, consequently, a high signal-to-noise ratio. Despite rigorously matching the words for factors known to affect memory (Table S1), positive and negative items still differed in intensity. Our P300 results, however, suggest that future studies need to consider intensity in order to obtain unambiguous results. Replications of our study could use languages, such as English or Spanish, which allow controlling for intensity with existing extensive word norms.

Fourth, source memory tasks are common in IC research, but might not be optimally suited for investigating the relationship between ICs and memory. Source memory tasks provide cues (i.e., the complete item without source information), which are not present during frequency estimation. Thus, participants might recognize items during test which they cannot retrieve during frequency estimation. Moreover, participants assign items to the majority or the minority in the source memory task, whereas they gauge the number of negative items for the majority and minority during frequency estimation (i.e., the reverse question). This situation might be aggravated in the present study by the sheer amount of items, because participants might have relied on small samples of the retrieved items during frequency estimation. Because free recall provides direct insight into what participants spontaneously retrieve from memory, it might provide a feasible alternative to the source memory task for investigating the relation between memory and IC. Moreover, there is a clear need for more research investigating how the relationship between memory performance and ICs change as the number of studied items increases.

## Conclusions

Our study makes three major contributions to the literature. First of all, our study is, to the best of our knowledge, the first to investigate the distinctiveness-based IC in an ERP paradigm using all essential features of the illusory correlation paradigm by Hamilton and Gifford (1976). We found an IC even after extended learning and thereby replicated prior research.

Second, our behavioral and ERP results are incompatible with the SDA, indicating that this account might not be suitable for explaining the formation of ICs. Rather, we propose AT, which encompasses both accentuation and distinctiveness in a unifying theory (Sherman et al., 2009), as one intriguing post-hoc explanation for the (from the perspective of the SDA) unexpected cross-over interaction pattern in the ERP and memory data. However, we could not link attention shifts to memory formation processes indexed by the P300 SME. Clearly, more research, especially with psychophysiological methods like ERPs or eye-tracking, is needed to establish how exactly attention allocation contributes to ICs. AT (Sherman et al., 2009) may provide an integrative perspective to guide future studies.

Third, the current study shows that emotional intensity is relevant for ICs. For the P300, a cross-over interaction was observed only for high intensity items. Thus, the P300 findings suggest that minority items become distinct because of their combination of rareness, negative valence, and intensity, rather than just rareness and negative valence. At the same time, our study shows that the IC can be observed even after controlling for arousal, concreteness, word length, and word frequency, indicating that these factors are not pivotal for illusory correlations to arise.

Our study blended the neuroscience of learning and memory with the literature on social cognition and provided new insights in the neural underpinnings of stereotype acquisition. However, more research is needed in order to obtain a more integrative view of social cognition and cognitive neuroscience.

## Electronic supplementary material

ESM 1 (DOCX 303 kb)

## Footnotes

1 Some might argue that study block should be treated as a level 1 variable rather than as a level 2 variable. Therefore, we conducted an additional multilevel analysis with study block as Helmert contrast coded level 1 variable. This model rendered virtually identical results as our three-level model. One noteworthy result from this analysis was that the P300 was lower in the first block relative to all other blocks. For the sake of clarity, we decided to use the three-level model for interpretation.
