# Supplementary material for: The effect of shared distinctiveness on source memory: An event-related potential study
Source: Cogn Affect Behav Neurosci. 2020 Aug 24;20(5):1027–40. doi: 10.3758/s13415-020-00817-1 (PMC7497493; doi:10.3758/s13415-020-00817-1)
Supplement: Supplementary file 1 — (DOCX 303 kb) [file 13415_2020_817_MOESM1_ESM.docx]

Supplementary Materials

**S1 Descriptive statistics for the stimulus material**

Table S1. Mean, standard deviation (in brackets), and inferential statistics for the positive and negative words. Please note that only the log-transformed word frequencies were subjected to statistical analysis.

|  | Positive  (N = 320) | Negative  (N = 160) | Comparison |
| --- | --- | --- | --- |
| Valence | 7.54 (0.53) | 2.90 (0.62) | F(1, 478) = 7298.08, p < .001 |
| Intensity | 2.54 (0.53) | 2.10 (0.62) | F(1, 478) = 65.52, p < .001 |
| Arousal | 4.67 (1.31) | 4.73 (1.21) | F(1, 478) = 0.25, p = .618 |
| Concreteness | 6.49 (1.81) | 6.38 (1.55) | F(1, 478) = 0.43, p = .510 |
| Word length | 7.49 (2.86) | 7.33 (2.54) | F(1, 478) = 0.39, p = .535 |
| Word frequency | 5701 (9180) | 3141 (4252) | - |
| Log(Word frequency) | 7.66 (1.59) | 7.56 (0.92) | F(1, 478) = 0.62, p = .431 |

**S2 Calculation of the unbiased hit rates**

Wagner (1993) proposed the unbiased hit rates as a measure of accuracy that corrects for response biases. In a 3 x 3 matrix (Table S2), unbiased hit rates for stimulus 1 (e.g. majority items) are calculated as follows:

$$H{}_{U}=\frac{a}{a+b+c} \cdot\frac{a}{a+d+g}$$

In a similar vein, the unbiased hit rates for stimulus 2 (e.g. minority items) are:

$$H{}_{U}=\frac{e}{d+e+f} \cdot\frac{e}{b+e+h}$$

and for stimulus 3 (e.g. new items):

$$H{}_{U}=\frac{i}{g+h+i} \cdot\frac{i}{c+f+i}$$

Table S2. A 3 x 3 matrix. Each entry represents the absolute frequency for this cell.

|  |  | Judgment |  |
| --- | --- | --- | --- |
| Stimulus | 1 (Majority) | 2 (Minority) | 3 (New) |
| 1 (Majority) | a | b | c |
| 2 (Minority) | d | e | f |
| 3 (New) | g | h | i |

**S3 Overview over the descriptive statistics on the trial numbers**

Table S3. Descriptive statistics on the trial numbers.

|  |  | Forgotten | | | | Remembered | | | |
| --- | --- | --- | --- | --- | --- | --- | --- | --- | --- |
|  |  | M | SD | Min. | Max. | M | SD | Min. | Max. |
| Majority | Positive | 15.4 | 11.1 | 0 | 50 | 63.4 | 11.2 | 30 | 78 |
|  | Negative | 7.5 | 6.2 | 1 | 29 | 31.8 | 6.2 | 10 | 39 |
| Minority | Positive | 15.2 | 10.5 | 0 | 47 | 63.5 | 10.5 | 30 | 70 |
|  | Negative | 7.4 | 6.3 | 0 | 27 | 32.1 | 6.6 | 13 | 40 |

**S4 Judgment of Learning**

In the present study, we implemented judgment of learning (JOL; e.g. Nelson & Narens, 1990) –the learner´s subjective judgment about the likelihood of remembering an item in a subsequent memory test – as an additional online measure of distinctiveness and a manipulation check for shared distinctiveness. Since the studies by Dunlosky et al.(2000) and Geraci and Manzano (2010) suggested that JOLs capture the meta-cognitive belief of participants that stimuli which “pop out” of their surrounding should also be better remembered, we hypothesized that negative minority items should receive higher JOLs than all other category combinations. Moreover, JOL represents a semantic encoding task, as recommended to be used for P300 SME studies (e.g. Fabiani & Donchin, 1995).

*Data analysis.* Distinctiveness as perceived during encoding was assessed by analyzing the JOLs. The JOLs were averaged for each category of items and compared in a 2 x 2 repeated-measure ANOVA with the factors Source (majority vs. minority) and Valence (positive vs. negative).

*Results and discussion.* The analysis of the JOLs (see Table S4 for descriptive statistics) revealed a significant main effect for valence (F(1, 39) = 15.956, p < .001, η_p_² = .290). Positive words received higher JOLs than negative words. Neither the main effect for source nor the interaction between source and valence were significant (F(1, 39) = 1.442, p = .237, η_p_² = .036 and F(1, 39) = .021, p = .885, η_p_² = .001, respectively). The JOL findings do not provide evidence that rare, negative items popped out, as initially presumed.

Table S4. Mean judgment of learning ratings (± SD) for positive and negative words.

|  |  | Majority | Minority |
| --- | --- | --- | --- |
| Overall | Positive | 4.14 (0.49) | 4.23 (0.63) |
|  | Negative | 3.80 (0.46) | 3.90 (0.65) |

Since the words were not matched for emotional intensity, we calculated a hierarchical linear model (HLM) analysis with the JOLs as criterion and the grand-mean centered variables Valence (Positive: 0.33, Negative: -0.67), Source, (Majority: 0.33, Minority, -0.67) and Intensity as predictors. As in the analysis of the P300, blocks and participants were used as random factors with blocks nested within participants. The full model was better than the model containing only the random intercepts (χ²(7) = 428.15, p < .001). As can be seen in Table S5, there was a main effect for Valence and Source indicating JOLs were higher for positive items than negative items and for minority items than for majority items. This essentially replicates the results from the repeated-measure ANOVA. In addition, there was a main effect for intensity indicating that the JOLs became higher with increasing intensity. This effect was modulated by Valence. The increase of JOLs as a function of intensity was higher for positive items as compared to negative items. This additional analysis also did not provide evidence that rare, negative items popped out. However, the results indicate that high intense items were perceived as more distinctive that low intense items.

Table S5. Information on model fit for the hierarchical linear model analyses.

|  | B | SE B | t(13993) |
| --- | --- | --- | --- |
| (Intercept) | 4.04 | 0.06 | 64.93 (p < .001) |
| Valence | 0.27 | 0.02 | 12.94 (p < .001) |
| Source | -0.09 | 0.02 | -4.41 (p < .001) |
| Intensity | 0.11 | 0.1 | 10.12 (p < .001) |
| Valence x Source | 0.02 | 0.04 | 0.27 (p = .784) |
| Valence x Intensity | 0.10 | 0.02 | 4.82 (p < .001) |
| Source x Intensity | -0.00 | 0.02 | -0.01 (p = .989) |
| Valence x Source x Intensity | 0.00 | 0.04 | 0.11 (p = .913) |

Table S6. Predicted JOLs. Please note that low, medium and high intensity were defined -1, 0 and +1 SD of the z-standardized, log-transformed intensity variable.

|  |  | Majority | Minority |
| --- | --- | --- | --- |
| Low intensity | Positive | 3.96 | 4.05 |
|  | Negative | 3.78 | 3.88 |
| Medium intensity | Positive | 4.10 | 4.19 |
|  | Negative | 3.82 | 3.92 |
| High intensity | Positive | 4.24 | 4.33 |
|  | Negative | 3.87 | 3.97 |

**S5 Frequency estimation for the last block**

We assessed frequency estimates not only for the whole session, but also for the last block, because few IC studies used more than 48 study trials and an IC might be observable in the frequency estimates for the last block (36 study trials), but not in the frequency estimates for the whole experiment (360 study trials).

The descriptive statistics for the frequency estimations of the last block can be found in Table S5. When participants made judgments for the last block, the frequency was not overestimated, (t(39) = -1.115, p = .136, one-sided, Cohen’s d = 0.18). However, participants correctly rated the majority source as highly frequent in the last block (t(39) = 4.03, p < .001, one-sided, Cohen’s d = 0.64). However, their estimates were lower than the actual frequency (0.67). We also calculated a phi coefficient from the frequency ratings of the last block and found a significant IC (t(39) = 1.16, p = 128., one-sided, Cohen’s d = 0.18). In a direct comparison between the estimates of the last block and the estimates for the whole experiment, a significant effect was only observed the frequency estimate for the negative minority. Thus, the frequency estimates did not substantially differ between conditions pointing to the stability of the IC.

Table S7. Descriptive and inferential statistics for the frequency estimation task for the last block and the whole session.

|  | Last block | | | | Whole session | | | | Comparison | |
| --- | --- | --- | --- | --- | --- | --- | --- | --- | --- | --- |
|  | M | | SD | | M | | SD | |  | |
| Negative majority words | .36 | .16 | | .39 | | .15 | | t(39) = -1.47, p = .151, Cohen’s d = -0.23 | |  |
| Negative minority words | .39 | .18 | | .46 | | .16 | | t(39) = -2.39, p = .022, Cohen’s d = -0.38 | |  |
| Overall frequency of the majority | .60 | .15 | | .61 | | .15 | | t(39) = -0.72, p = .477, Cohen’s d = -0.11 | |  |
| Phi coefficient | .04 | .22 | | .07 | | .21 | | t(39) = -1.00, p = .324, Cohen’s d = 0.16 | |  |

**S6 Additional analysis for source confidence judgments**

In order to control for the effect of emotional intensity, we calculated a hierarchical linear model (HLM) with the source confidence as criterion and the grand-mean centered variables Valence (Positive: 0.33, Negative: -0.67), Source, (Majority: 0.50, Minority, -0.50) and Intensity as predictors. As in the analysis of the P300, blocks and participants were used as random factors with blocks nested within participants. The full model was better than the model containing only the random intercepts (χ²(7) = 132.82, p < .001). As can be seen in Table S7, there was a main effect Source indicating confidence was higher for minority items than for majority items. Furthermore, there was a significant interaction between Valence and Source. This essentially replicates the results from the repeated-measure ANOVA in the main text. In addition, there was a main effect for intensity indicating that the confidence became higher with increasing intensity. No additional effects or interactions were observed. This additional analysis revealed that emotional increased the confidence in the source judgment, but did not alter the relationships we reported in the main text.

Table S8. Information on model fit for the hierarchical linear model analyses.

|  | B | SE B | t(7341) |
| --- | --- | --- | --- |
| (Intercept) | 4.34 | 0.07 | 60.83 (p < .001) |
| Valence | 0.05 | 0.04 | 1.31 (p = .191) |
| Source | 0.27 | 0.03 | 7.95 (p < .001) |
| Intensity | 0.04 | 0.02 | 2.30 (p = .021) |
| Valence x Source | 0.45 | 0.07 | 6.32 (p < .001) |
| Valence x Intensity | 0.05 | 0.03 | 1.36 (p = .173) |
| Source x Intensity | -0.00 | 0.04 | -0.11 (p = .910) |
| Valence x Source x Intensity | 0.04 | 0.07 | 0.53 (p = .595) |

Table S9. Predicted confidence in source judgment. Please note that low, medium and high intensity were here defined -1, 0 and +1 SD of the z-standardized, log-transformed intensity variable.

|  |  | Majority | Minority |
| --- | --- | --- | --- |
| Low intensity | Positive | 4.50 | 4.10 |
|  | Negative | 4.30 | 4.30 |
| Medium intensity | Positive | 4.57 | 4.15 |
|  | Negative | 4.29 | 4.33 |
| High intensity | Positive | 4.63 | 4.20 |
|  | Negative | 4.29 | 4.35 |

**S7 Additional ERP figures**

Figure S1. P300 for forgotten and remembered items.

Figure S2. P300 for majority and minority items.

Figure S3. P300 for positive and negative items.

Figure S4. P300 for forgotten and remembered items at high, medium, or low intensity levels.

Figure S5. P300 for forgotten and remembered majority and minority items. The color purple denotes the majority and the color orange denotes the minority. Solid lines denote remembered items and dashed lines denote forgotten items.

**References**

Dunlosky, J., Hunt, R. R., & Clark, E. (2000). Is perceptual salience needed in explanations of the isolation effect? *Journal of Experimental Psychology: Learning, Memory, and Cognition*, *26*(3), 649–657. https://doi.org/10.1037/0278-7393.26.3.649

Fabiani, M., & Donchin, E. (1995). Encoding processes and memory organization: A model of the von Restorff effect. *Journal of Experimental Psychology: Learning, Memory, and Cognition*, *21*(1), 224–240. https://doi.org/10.1037/0278-7393.21.1.224

Geraci, L., & Manzano, I. (2010). Distinctive items are salient during encoding: Delayed judgements of learning predict the isolation effect. *The Quarterly Journal of Experimental Psychology*, *63*(1), 50–64. https://doi.org/10.1080/17470210902790161

Nelson, T. O., & Narens, L. (1990). Metamemory: A Theoretical Framework and New Findings. In G. H. Bower (Ed.), *Psychology of Learning and Motivation* (Vol. 26, pp. 125–173). Academic Press. https://doi.org/10.1016/S0079-7421(08)60053-5

Wagner, H. L. (1993). On measuring performance in category judgment studies of nonverbal behavior. *Journal of Nonverbal Behavior*, *17*(1), 3–28. https://doi.org/10.1007/BF00987006
